# Effect of typhaneoside on ventricular remodeling and regulation of PI3K/Akt/mTOR pathway

**DOI:** 10.1007/s00059-019-4819-2

**Published:** 2019-06-14

**Authors:** X. Zhang, K. Yang, H. Zhang, W. Dong, W. Peng, Y. Zhao

**Affiliations:** 1 Department of Cardiology and Nephrology, The No. 1 Hospital of PLA, 730030 Lanzhou, China; 2 Department of Cardiac Surgery Intensive Care Unit, The Second hospital of Lanzhou university, 730030 Lanzhou, China

**Keywords:** Traditional Chinese medicine, Heart failure, Myocardial infarction, Autophagy, Transduction, Traditionelle chinesische Medizin, Herzinsuffizienz, Myokardinfarkt, Autophagie, Transduktion

## Abstract

**Background:**

This study aimed to investigate the effect of typhaneoside on ventricular remodeling and regulation of the PI3K/Akt/mTOR autophagy transduction pathway in rats with heart failure after myocardial infarction.

**Methods:**

The effects of typhaneoside on the general condition of rats were observed in vivo using a rat model of heart failure after myocardial infarction had been established. The expression of serum N‑terminal pro-brain natriuretic peptide (NT-proBNP), matrix lysin 2 (ST2), interleukin-6 (IL-6), tumor necrosis factor alpha (TNF-α), matrix metalloproteinase 2 (MMP-2), and MMP-9 was detected via ELISA. A hypoxia/reoxygenation model was established to analyze the number and morphology of autophagosomes in vitro by transmission electron microscopy. Light chain 3 (LC3) variations were detected by immunofluorescence. Western blotting was used to assess LC3-II/LC3-I and p62 expression as well as p‑Akt/Akt, p‑mTOR/mTOR ratios.

**Results:**

Compared with the sham group, the general condition scores of the rats in the model group decreased significantly, while the expression of serum NT-proBNP, ST2, IL-6, TNF-α, MMP-2, and MMP-9 increased. The number of autophagosomes in the drug-containing serum group was significantly reduced and the ratio of LC3-II/LC3-I was significantly decreased. The expression of P62 protein was increased, and the ratios of p‑Akt/Akt and p‑mTOR/mTOR were significantly increased.

**Conclusion:**

Typhaneoside regulates IL-6 and TNF-α as well as MMP-2 and MMP-9 in rats with heart failure after myocardial infarction. Typhaneoside can improve cardiac morphological structure and myocardial remodeling and enhance heart function. It may mediate autophagy inhibition in the cardiomyocyte anoxia/reoxygenation (A/R) pathway through the PI3K/Akt/mTOR autophagy transduction pathway.

## Abstract (de)

**Hintergrund:**

Ziel der vorliegenden Studie war, die Wirkung von Typhaneosid auf ventrikuläres Remodeling und die Regulierung des PI3K/Akt/mTOR-Autophagie-Transduktions-Signalwegs bei Ratten mit Herzinsuffizienz nach Myokardinfarkt zu untersuchen.

**Methoden:**

An einem Rattenmodell der Herzinsuffizienz nach Auslösen eines Myokardinfarkts wurden die Wirkungen von Typhaneosid auf den Allgemeinzustand von Ratten in vivo beobachtet. Mittels ELISA wurde die Expression von NT-ProBNP („N-terminal pro-brain natriuretic peptide“), Matrixlysin 2 (ST2), Interleukin-6 (IL-6), Tumornekrosefaktor-α (TNF-α), Matrixmetalloproteinase-2 (MMP-2) und MMP-9 im Serum bestimmt. Ein Modell der Hypoxie/Reoxygenierung wurde entwickelt, um per Transmissionselektronenmikroskopie Anzahl und Morphologie von Autophagosomen in vitro zu ermitteln. Durch Immunfluoreszenz wurden Varianten der leichten Kette 3 (LC3) nachgewiesen. Das Westernblot-Verfahren wurde eingesetzt, um die Expression von LC3-II/LC3-I und p62 sowie der Quotienten p‑Akt/Akt und p‑mTOR/mTOR zu ermitteln.

**Ergebnisse:**

Im Vergleich zur Gruppe mit Scheinbehandlung verschlechterte sich der Allgemeinzustand der Ratten in der Modellgruppe signifikant, während die Expression von NT-proBNP, ST2, IL-6, TNF-α, MMP-2 und MMP-9 im Serum zunahm. Die Anzahl der Autophagosomen in der Gruppe, deren Serum das Medikament enthielt, war signifikant vermindert, ebenfalls der Quotient LC3-II/LC3-I. Es bestand eine vermehrte Expression des Proteins P62, und die Quotienten p‑Akt/Akt und p‑mTOR/mTOR zeigten sich signifikant erhöht.

**Schlussfolgerung:**

Typhaneosid reguliert IL-6 und TNF-α sowie MMP-2 und MMP-9 bei Ratten mit Herzinsuffizienz nach Myokardinfarkt. Unter Typhaneosid können sich die morphologische Struktur des Herzens und das myokardiale Remodeling verbessern sowie die Herzfunktion steigern. Möglicherweise ist es Mediator der Autophagiehemmung im A/R-Signalweg der Kardiomyozyten durch den PI3K/Akt/mTOR-Autophagie-Transduktions-Signalweg.

Heart failure (HF) is a frequently encountered condition in clinical practice; however, it is difficult to qualify its incidence. Ventricular remodeling is an important pathological process in HF. How to control or even reverse ventricular remodeling and thereby improve heart function has been a hot topic in clinical research [1].

Heart failure is the ultimate outcome of a variety of cardiovascular diseases, developing in a progressive manner. Clinically, even if the patient’s symptoms have not progressed, the natural progression of HF continues to occur. During this process, a series of changes in molecular and cellular mechanisms trigger alterations in myocardial structure, function, and phenotype [2], which is also manifested by pathological hypertrophy of cardiomyocytes, apoptosis, and excessive fibrosis or excessive degradation of the extracellular matrix. At the same time, due to the increased excitability of the neuroendocrine renin–angiotensin system (RAS system) and the sympathetic nervous system, a large number of neuroendocrines and cytokines are activated. After the activation of the RAS system and cytokines, ventricular remodeling is further aggravated, and myocardial damage and deterioration of cardiac function are further exacerbated. The deterioration in cardiac function has an effect on the RAS system and cytokines, and in this vicious circle, HF continues to be aggravated [3, 4].

Autophagy has been widely found in cardiomyocytes in HF as a result of dilated cardiomyopathy, valvular heart disease, and ischemic heart disease [5]. Autophagy is closely related to HF, but the mechanism needs to be further examined and clarified. Autophagy is an important process of cell metabolism, and the improvement of ventricular remodeling through the regulation of the autophagy pathway is also a central topic of research. The phosphoinositide-3-kinase/protein kinase B/mammalian target of rapamycin (PI3K/Akt/mTOR) pathway, a classic pathway of autophagy, plays an important role in the pathogenesis of HF [6, 7].

Typhaneoside has certain effects on the cardiovascular system, including lowering blood lipid levels, promoting anti-atherosclerosis activities, activating the uterine and intestinal smooth muscle, as well as improving immune and coagulation function. However, to date, there has been scant research and few reports on the treatment of HF with this traditional Chinese medicine. The present study, thus, has innovative significance.

## Methods

### In vivo experiments

#### Model establishment and animal grouping

A total of 150 specific-pathogen-free (SPF) male Sprague–Dawley rats, 8 weeks old, weighing 200 ± 20 g, were obtained. After fasting for 12 h before surgery, the rats were provided with free drinking water. Using a noninvasive inhalation anesthesia system, with the ventilation rate set to 1.5 l/min, the rats inhaled 2% isoflurane. An electrocardiogram (II lead) was then recorded. The left anterior descending coronary artery was ligated. The rats were placed on the test bench; at 2 cm to the intercostal space between the fourth and fifth left sternum, a 2-cm longitudinal incision was made with ophthalmic scissors, and the pectoralis major and the chest were bluntly separated. A broken purse-string suture was made around the incision, and the suture was temporarily not tightened.

With the help of a hemostat, the pleura and pericardium were penetrated at the fourth intercostal space, the chest was opened and the left chest wall was pressed clockwise. The apex was then opened toward the chest wall, and the heart was squeezed out of the chest with the index finger and thumb of the left hand. At 2 cm to the lower edge of the left atrial appendage, the left coronary artery was ligated with a small round needle and a 6/0 nonabsorbable line. The apex instantly turned white, and the heart was quickly placed in the chest cavity; the purse-string suture was tightened, the chest wall was squeezed to remove thoracic gas, and the chest cavity was closed.

In the sham group, 20 rats were treated with the same surgical procedure, but the anterior descending artery was only threaded without ligation. Serum troponin I levels were measured 24 h after surgery to determine whether the myocardial infarction model was successfully established. Postoperatively, 200,000 u/kg penicillin via an intraperitoneal injection was routinely administered for 7 days as anti-inflammatory treatment. The animals received routine feeding for 4 weeks. After the myocardial infarction model was established, the rats were included in the experiments.

The 16 live rats in the sham group (group A) were given the same dose of normal saline. The 80 successfully modeled rats were randomly divided into four groups: the model group (group B) was given the same dose of normal saline; the typhaneoside low-dose group (group C) was given 10 mg/kg of typhaneoside; he typhaneoside middle-dose group (group D) was given 20 mg/kg of typhaneoside; the typhaneoside high-dose group (group E) was given 40 mg/kg of typhaneoside. The typhaneoside infusion was administered once a day for 4 weeks.

#### Ultrasound examination

After 4 weeks of treatment, the left ventricular short axis shortening rate (LVFS), stroke volume (SV), heart rate (HR), cardiac output (CO), left ventricular end diastolic diameter (LVEDD), left ventricular end systolic diameter (LVESD), and left ventricular ejection fraction (LVEF) were all measured.

#### Hemodynamic testing

After 4 weeks of treatment, mean arterial pressure (MAP), left ventricular diastolic pressure (LVDP), left ventricular systolic pressure (LVSP), left ventricular end diastolic pressure (LVEDP), left ventricular maximum ascending rate (+dp/dtmax), and the maximum rate of decrease in left ventricular pressure (−dp/dtmax) were all determined.

#### ELISA analysis

Enzyme-linked immunosorbent assay (ELISA) was used to detect the expressions of serum N‑terminal pro-brain natriuretic peptide (NT-proBNP), matrix lysin 2 (ST2), interleukin-6 (IL-6), tumor necrosis factor alpha (TNF-α), matrix metalloproteinase 2 (MMP-2), and MMP-9. The aluminum foil bag with slats was equilibrated at room temperature for 20 min, and the required strips were removed. Standard holes, sample holes, and blank holes were set on the strips; the blank holes were not filled with any liquid, while the standard and sample holes were filled with different concentrations of 50-μl samples. A total of 10 ml sample was added to the holes for testing, and then a 40-ml sample dilution was added. Subsequently, 50 ml horseradish peroxidase-labeled detection antibody was added to the standard and the sample holes, which were sealed with a film and placed in an incubator at 37 °C. The incubation time was set to 60 min. The liquid was discarded and the required strips patted dry on the absorbent paper. Washing liquid was injected into the hole. After 1 min, the washing liquid was removed and the strips then placed on the absorbent paper to dry; this was repeated five times. Substrate A and B at 50 ml were added to each hole, respectively, and then placed in an incubator at 37 °C in darkness. Stop solution 50 μl was added to each hole, and the optical density (OD) value at a wavelength of 450 nm was measured within 15 min.

#### Western blot

Tissue was collected from each group. A total of 400 μl cell lysate was added, followed by 40 μl of phenylmethylsulfonyl fluoride (PMSF), and the cell culture flask was gently shaken and placed on ice for 10 min to lyse the cells evenly. The cells were repeatedly aspirated with a sterile syringe, and the lysed product was collected in an Eppendorf (EP) tube and placed in an ice bath for 30 min. The cells were centrifuged at 12,000 *g* for 15 min. The supernatant was transferred to a new EP tube, and 100 μl of each tube was added to 20 μl of protein-loaded buffer. The cells were boiled for 5 min, mixed, and then stored at −80 °C. Samples were obtained and the proteins were separated by 12% SDS-PAGE electrophoresis. The separated protein bands were transferred to the PVDF membrane using the wet method, and then sealed at room temperature for 1 h. Following this, the primary antibody was added (light chain 3 [LC3]-II/I, p62, Akt, mTOR [concentration 1:1000]). The cells were incubated overnight at 4 °C. The primary antibody was eluted, and the secondary antibody (1:1000) was added for 1 h of incubation. The secondary antibody was then washed off. Color development and fixation were completed for chemiluminescence. The expression of PI3K/Akt/mTOR signaling pathway-associated proteins was measured.

### In vitro experiments

#### Primary neonatal rat cardiomyocyte culture

The skin of the chest wall of the suckling rat was disinfected, the chest cavity was cut open along the left edge of the xiphoid process of the sternum, the heart was fully exposed, and the apex was clamped with ophthalmology scissors. The heart was then bluntly separated and placed in a Petri dish containing pre-cooled phosphate buffered saline (PBS). The heart was rinsed thoroughly three times. The pericardium, large blood vessels, and atrial tissue around the heart were removed. The ventricular muscles were cut into 1–3-mm tissue pieces and then placed in a serum bottle. An equal volume of type I collagenase (0.08%) and trypsin (0.08%) was placed in a serum bottle, then gently pipetted and mixed at 37 °C for 5‑min digestion in a magnetic stirrer (200 rpm). The first aspirated supernatant was discarded, and the digestion was continued for 5 min. Following this, the aspirated supernatant was added to the serum-containing medium to terminate the digestion, and the digested product was filtered through a 200-mesh filter until the tissue block disappeared or the supernatant was clear. The filtrate was centrifuged on a horizontal centrifuge (1000 rpm) for 5 min, the supernatant was removed, and the pellet was fully resuspended in the complete medium. The cells were suspended in a Petri dish and placed in a cell culture incubator with 5% CO_2_ at 37 °C, and then subjected to differential adherence. Following this, the unattached cardiomyocyte suspension was aspirated by pipetting the bottom of the culture dish, and then placed in a complete medium containing 20% fetal bovine serum, after which the cells were counted with a cell-counting plate. The cells were inoculated into different cell culture plates at a density of 5 × l0^5^/ml. The cells were added to 0.1 mol/l Brdu and placed in a cell culture incubator with 5% CO_2_ at 37 °C. Pulsation was observed for a small portion of cells after 24 h, and the cells were basically adherent after 48 h. The medium was changed every 2–3 days according to the metabolic state of the cells.

#### Preparation of drug-containing serum

We randomly divided 60 male Sprague-Dawley rats into two groups: a drug-administered group and a control group, with 30 rats in each group. The drug-administered group was given 40 mg/kg of typhaneoside, while the control group was given an equal volume of saline. Rats were routinely fed for 4 days, with fasting on the fourth day after the last gavage. Saline or typhaneoside was administered to the rats twice on the fifth day, with a 1-h gap between administration, and blood samples were obtained 1 h after the last gavage. After anesthetizing the rats with 3% pentobarbital, blood was obtained from the abdominal aorta of the rats with a negative-pressure blood collection needle, and the group number was indicated. The blood sample was centrifuged at 3000 rpm at room temperature for 15 min. After pipetting the supernatant, the blood was placed in a water bath for 30 min and the temperature was set to 56 °C. After deactivation, the cells were sterilized by filtration using a 0.22-μm microporous membrane. Following this, the blood was stored in 2‑ml sterile EP tubes.

#### Hypoxia–reoxygenation model

After pretreatment of primary cardiomyocytes with drug-containing serum, the medium was removed, the cells were washed once with PBS, then PBS was added again, and the cells were placed in an oxygen-deficient box. Nitrogen was replaced with oxygen in the hypoxia box, and the box was sealed. The box was placed in an incubator at 37 °C, with hypoxia conditions for 12 h. Following this, the cells were removed from the hypoxia box, the PBS was removed and replaced with the complete medium, and the cells were cultured in a constant temperature box at 37 °C, with reoxygenation for 4 h. The experimental grouping was as follows:

A. The control group—48 h after adherence, the myocardial cells were incubated for 17 h.
B. The model (hypoxia/reoxygenation [A/R]) group— 48 h after adherence, the myocardial cells were incubated for 1 h, with hypoxia for 12 h and reoxygenation for 4 h.
C. The drug + A/R group—48 h after adherence, the myocardial cells were incubated for 0.5 h, then 40 mg/kg drug-containing serum was added for 0.5-h pretreatment, with hypoxia for 12 h, and reoxygenation for 4 h.
D. The rapamycin (RA) + A/R group—48 h after adherence, the myocardial cells were normally incubated for 0.5 h; RA was added for 0.5-h pretreatment, with hypoxia for 12 h and reoxygenation for 4 h.
E. The drug + RA + A/R group—48 h after adherence, the myocardial cells were incubated for 0.5 h, then drug was added for 0.5-h pretreatment, with hypoxia for 12 h and reoxygenation for 4 h.
F. The API-2 + A/R group—48 h after adherence, the myocardial cells were normally incubated for 0.5 h, then drug was added for 0.5 h pretreatment, with hypoxia for 12 h and reoxygenation for 4 h.
G. The drug + API-2 + A/R group—48 h after adherence, the myocardial cells were added to API-2 for 0.5-h incubation, then drug was added for 0.5-h pretreatment, with hypoxia for 12 h and reoxygenation for 4 h.

The A, B, and C groups were prepared for electron microscopy and immunofluorescence, and the A, B, C, D, E, F, and G groups were prepared for Western blot analysis.

#### Electron microscopy

Glutaraldehyde (2.5%) was prepared with phosphate buffer. The tissue samples were fixed and rinsed with 0.1 M phosphate for 15 min, three times, and then fixed with 2% citrate fixative for 1 h. The tissue samples were rinsed with 0.1 M phosphate solution for 15 min, three times. Following this, the samples were placed in different concentrations of ethanol (50%, 70%, and 90%) and acetone (90%) for 15 min, then placed in 100% acetone at room temperature three times, for 15–20 min each time. The tissue samples were then placed in a solution with a 2:1 ratio of acetone to embedding solution for 3–4 h. Following this, the tissue samples were placed in a solution with a 1:2 ratio of acetone to embedding solution and left overnight. The tissue samples were placed in 37 °C pure embedding solution for 2–3 h. After the tissue samples were embedded, they were placed in an oven at 37 °C for 24 h, then in an oven at 45 °C for 12 h, and successively in an oven at 60 °C for 24 h. The tissue was sliced into 50–70-mm slices on a microtome. The slices were double-stained with uranyl acetate-lead citrate (3%) and prepared for transmission electron microscopy for observation.

#### Immunofluorescence detection of LC3

The paraffin slices were dewaxed in xylene I for 10 min, and then in xylene II for 10 min. The slices were successively rinsed in 100% xylene I, xylene II, and 80% alcohol for 10 min, respectively. The slices were then placed on a shaker and rinsed thoroughly with PBS for 5 min. After the antigen was repaired, the slices were naturally cooled at room temperature for 20–30 min, then placed on a shaker and rinsed thoroughly with PBS buffer for 3 min three times. The slices were thoroughly rinsed with PBS for 3 min three times, and 50 μl of primary antibody (LC3B) was added dropwise . The mixture was then incubated for 60 min; the incubator temperature was set to 37 °C. Subsequently, the slices were thoroughly rinsed with PBS for 3 min three times. The secondary antibody was added dropwise and the slices were incubated at room temperature for 60 min. The slices were thoroughly rinsed with PBS for 3 min three times, and then stained with DAPI for about 2 min. Following this, the slices were thoroughly rinsed with PBS for 3 min and observed under a fluorescence microscope.

#### Western blot

Cells from each group were collected. A total of 400 μl cell lysate was added, followed by 40 μl of PMSF, the cell culture flask was then gently shaken and placed on ice for 10 min to lyse the cells evenly. The cells were repeatedly aspirated with a sterile syringe, and the lysed product was placed in the EP tube, which was placed in an ice bath for 30 min. The cells were centrifuged at 12,000 *g* for 15 min. The supernatant was transferred to a new EP tube, and 100 μl of each tube was added to 20 μl of protein-loaded buffer. The cells were boiled for 5 min, mixed, and then stored at −80 °C. Samples were obtained, and the proteins were separated by 12% SDS-PAGE electrophoresis. The separated protein bands were transferred to the PVDF membrane using the wet method, then sealed at room temperature for 1 h. Following this, the primary antibody was added (LC3-II/I, p62, Akt, mTOR [concentration 1:1000]), and the cells were incubated overnight at 4 °C. The primary antibody was eluted and the secondary antibody (1:1000) was added for 1 h of incubation. The secondary antibody was washed off. Color development and fixation were completed for chemiluminescence. Finally, the expression of PI3K/Akt/mTOR signaling pathway-associated proteins was measured.

### Statistical analysis

All data are expressed as mean ± standard deviation (mean ± SD). The *t* test was used for comparison between two groups, and comparisons between more than two groups were performed using one-way analysis of variance (ANOVA). Statistical significance was set at *p* < 0.05. The data were analyzed using GraphPad Prism 5.0 (GraphPad Software, San Diego, CA, USA).

## Results

### Serum troponin I level and effect of treatment on cardiac function

The average level of serum troponin I in the sham-operated group was 0.91 ± 0.18 ng/ml and that in the model group was 4.23 ± 0.32 ng/ml (*p* < 0.05). The level of serum troponin I in the model group was higher than that in the sham-operated group.

After 4 weeks of drug treatment, compared with the sham group, the values of LVEDD, LVESD, LVEF, LVFS, SV, and CO in the model group decreased significantly. However, after the medication, the indexes changed significantly (LVEDD and LVESD decreased, LVEF, LVFS, SV, and CO increased), meaning the drug treatment improved the cardiac function of the rats, as shown in Table 1.

**Table 1:** Effect of treatment on cardiac function of rats

	LVEDD (mm)	LVESD (mm)	LVEF (%)	LVFS (%)	SV (ml)	CO (l/min)
Sham	6.52 ± 0.91**	3.91 ± 0.22**	79.34 ± 2.75**	41.00 ± 9.21**	0.28 ± 0.01**	167.44 ± 12.88**
Model	8.82 ± 0.43	6.66 ± 0.56	41.66 ± 4.44	20.82 ± 3.29	0.18 ± 0.03	53.89 ± 7.83
10 mg/kg	8.15 ± 0.77	6.33 ± 0.87	47.61 ± 8.31	21.22 ± 1.89	0.22 ± 0.09	136.9 ± 12.45*
20 mg/kg	7.93 ± 0.12*	5.73 ± 0.83	55.28 ± 5.41*	24.65 ± 3.83*	0.22 ± 0.05	142.35 ± 10.31**
40 mg/kg	7.45 ± 1.02**	4.83 ± 0.14**	59.88 ± 3.90**	29.92 ± 4.55*	0.25 ± 0.07*	153.28 ± 5.92**

*LVEDD* left ventricular end diastolic diameter, *LVESD* left ventricular end systolic diameter, *LVEF* left ventricular ejection fraction, *LVFS* left ventricular short axis shortening rate, *SV* stroke volume, *CO* cardiac output

**p< 0.01 vs. model

*p < 0.05 vs. model

### Effects of treatment on hemorheological parameters

Compared with the sham group, the values of MAP, LVDP, LVSP, LVEDP, +dp/dmax, and −dp/dtma in the model group were significantly reduced. After drug administration, all indexes were significantly different from those in the model group (see Table 2).

**Table 2:** Effects of treatment on hemorheological parameters

	MAP (mm Hg)	LVDP (mm Hg)	LVSP (mm Hg)	LVEDP (mm Hg)	+dp/dmax (mm Hg/s)	−dp/dtmax (mm Hg/s)
Sham	222.34 ± 14.98**	225.91 ± 9.35**	249.09 ± 4.02**	100.03 ± 2.94**	3988.98 ± 100.45**	3955.12 ± 139.01**
Model	179.40 ± 10.72	177.93 ± 9.82	180.63 ± 10.45	121.94 ± 8.72	2494.23 ± 104.56	2480.78 ± 134.89
10 mg/kg	188.83 ± 13.66	198.68 ± 4.92*	198.62 ± 2.93*	107.54 ± 6.62*	2687.89 ± 129.76**	2683.03 ± 111.94**
20 mg/kg	201.39 ± 11.30*	207.69 ± 13.65*	214.73 ± 8.87**	103.56 ± 9.03**	3156.11 ± 99.43**	3076.54 ± 109.73**
40 mg/kg	209.11 ± 12.03**	213.84 ± 4.82**	221.30 ± 7.65**	101.76 ± 4.44**	3334.76 ± 145.09**	3000.62 ± 98.89**

*MAP* mean arterial pressure, *LVDP* left ventricular diastolic pressure, *LVSP* left ventricular systolic pressure, *LVEDP* left ventricular end-diastolic pressure, *+dp/dtmax* left ventricular maximum ascending rate, *−dp/dtmax* maximum rate of decrease in left ventricular pressure

**p < 0.01 vs. model

*p < 0.05 vs. model

### Effects of treatment on serum NT-proBNP, ST2, IL-6, TNF-α, MMP-2, and MMP-9 expression

**Fig. 1:**
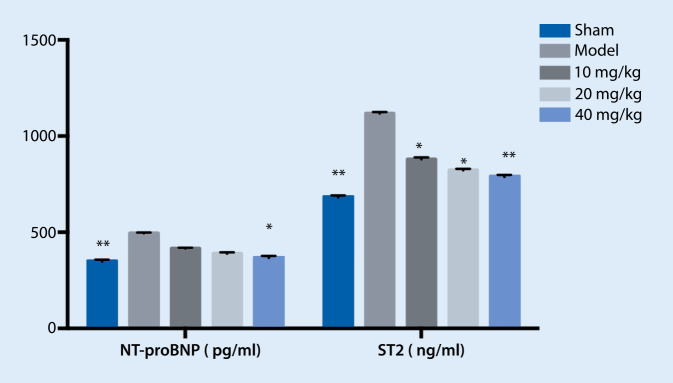
Effect of treatment on N‑terminal pro-brain natriuretic peptide (*NT-proBNP*) and matrix lysin 2 (*ST2*) expression. ** *p* < 0.01 vs. model, * *p* < 0.05 vs. model

**Fig. 2:**
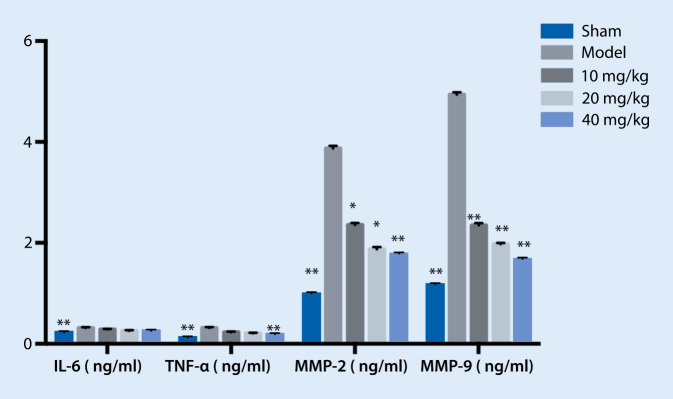
Effect of treatment on the expression of interleukin-6 (*IL-6*), tumor necrosis factor alpha (*TNF-α*), matrix metalloproteinase-2 (*MMP-2*), and matrix metalloproteinase-9 (*MMP-9*). ** *p* < 0.01 vs. model, * *p* < 0.05 vs. model

The expression of serum NT-proBNP, ST2, IL-6, TNF-α, MMP-2, and MMP-9 in the model group was significantly higher than that in the sham group, but it decreased after medication, as shown in Figs. 1 and 2.

### Transmission electron microscopy

**Fig. 3:**
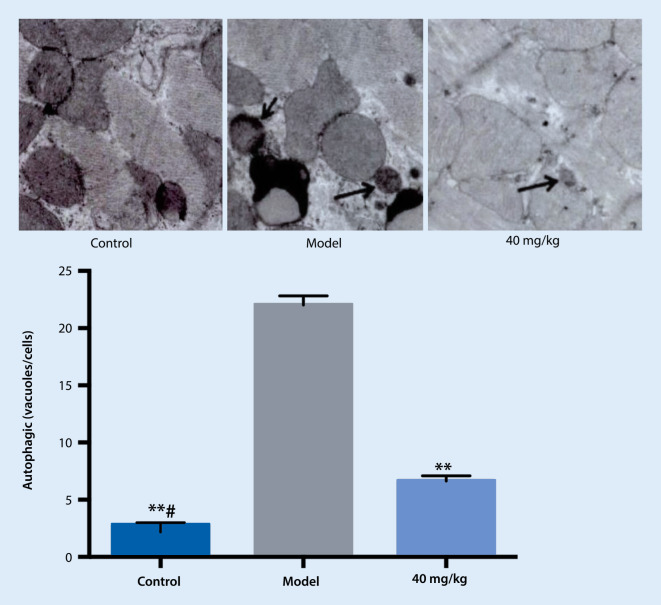
Effect of treatment on autophagosomes. ** *p* < 0.01 vs. model, # *p* < 0.05 vs. 40 mg/kg

The cell membrane and nuclear membrane of the control group were intact. The mitochondria and endoplasmic reticulum were normal and there were no autophagosomes in the cytoplasm. Compared with the control group, the cytoplasmic bilayer membrane autophagosome in the A/R group was significantly increased. Compared with the model group, the autophagosomes were reduced in the drug treatment group (see Fig. 3).

### Effects of different drug concentrations on proteins

**Fig. 4:**
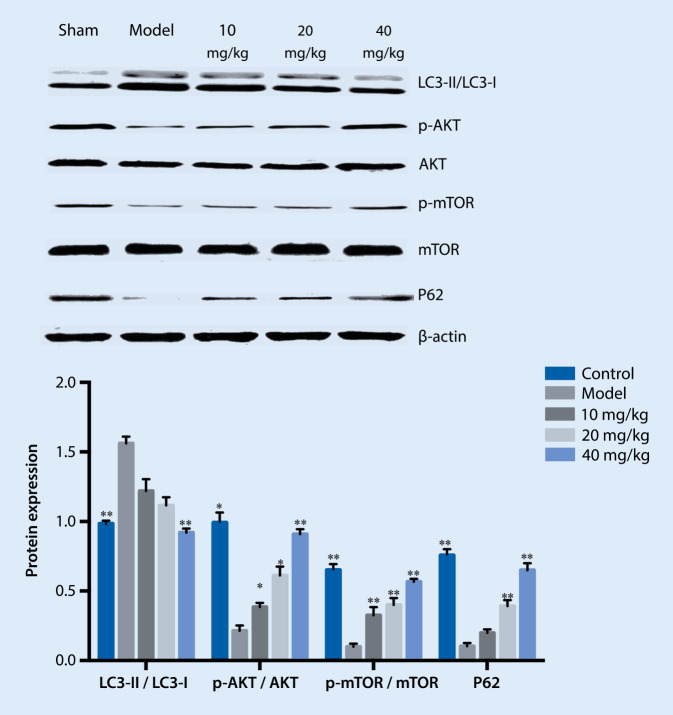
Effects of different concentrations of drug treatment on various proteins. ** *p* < 0.01 vs. model, * *p* < 0.05 vs. model. *LC3* light chain 3

Compared with the sham group, the LC3-II/LC3-I ratio increased and Akt/Akt, p‑mTOR/mTOR signaling decreased in the model group, while LC3-II/LC3-I decreased in the low-, medium-, and high-dose group and Akt/Akt, p‑mTOR/mTOR signaling increased, as shown in Fig. 4.

### Immunofluorescence detection of autophagy levels

**Fig. 5:**
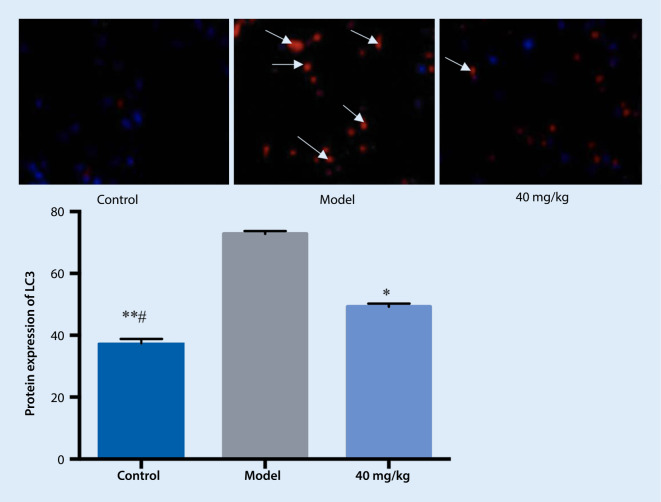
Effect of treatment on light chain 3 (*LC3, arrows*) expression in primary cardiomyocytes. ** *p* < 0.01 vs. model, * *p* < 0.05 vs. model, # *p* < 0.05 vs. 40 mg/kg

The blue fluorescence of the control group was evenly distributed in the cytoplasm, and there were almost no red fluorescent spots, indicating that there were few autophagosomes. Compared with the control group, the blue fluorescence spots were reduced in the cytoplasm of the model group, while the red fluorescent spots were increased. This suggested the presence of a large number of autophagosomes, indicating an increase in autophagy. Compared with the model group, the red fluorescent spots in the treatment group were reduced, implying that typhaneoside can reduce the formation of autophagosomes (see Fig. 5).

### Detection of PI3K/Akt/mTOR signaling pathway-associated protein expression

**Fig. 6:**
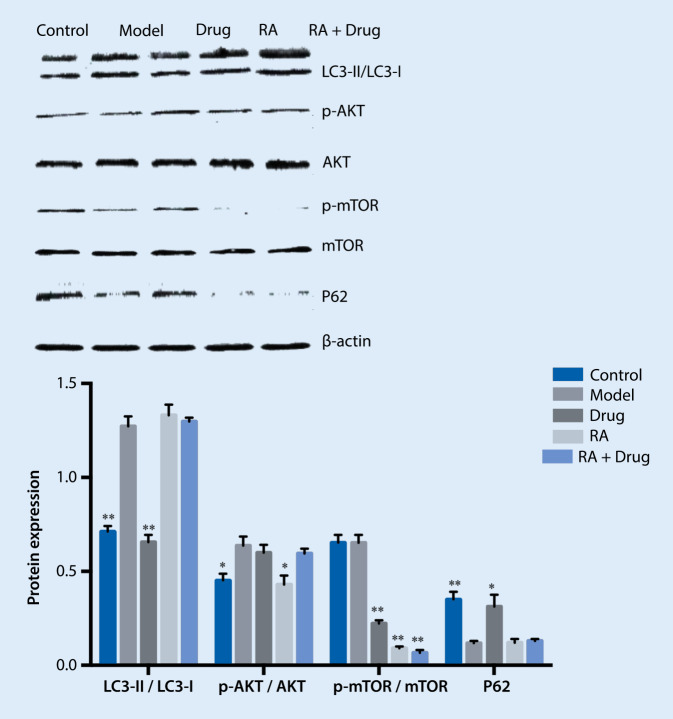
Effect of treatment on rapamycin (*RA*) pre-treated hypoxia/reoxygenation-induced myocardial autophagy. ** *p* < 0.01 vs. model, * *p* < 0.05 vs. model. *LC3* light chain 3. *RA* rapamycin

The results showed that, compared with the control group, the ratio of p‑mTOR/mTOR in the model group increased significantly. While compared with the model group, the ratio of p‑mTOR/mTOR in the treatment group was further improved. Compared with the model group, the p‑mTOR in the RA group decreased significantly. Compared with the RA group, the ratio of p‑mTOR/mTOR in the RA+ drug + A/R group decreased significantly, indicating that the drug treatment could not reactivate mTOR proteins after RA inhibited mTOR. This suggested that RA can block the autophagy inhibition of drugs in the hypoxia/reoxygenation process of cardiomyocytes, indicating that the drug-containing serum inhibits autophagy through the mTOR pathway, as shown in Fig. 6.

**Fig. 7:**
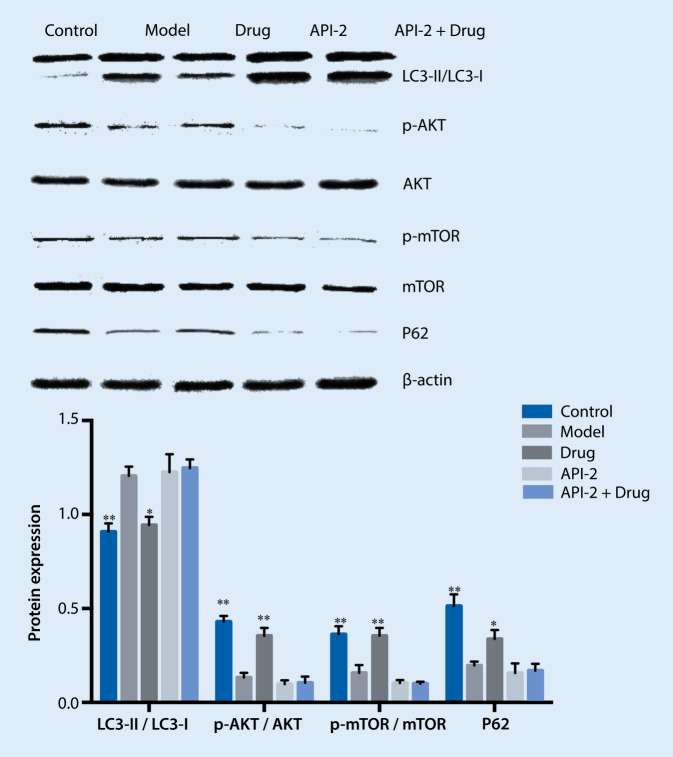
Effect of treatment on API-2 pre-treated hypoxia/reoxygenation-induced myocardial autophagy. ** *p* < 0.01 vs. model, * *p* < 0.05 vs. model. *LC3* light chain 3, *RA* rapamycin

Compared with the model group, the ratio of p‑Akt/Akt in the API-2 group decreased significantly. Compared with the drug + A/R group, the ratio of p‑Akt/Akt in the API-2+ drug + A/R group increased significantly. There was no significant change in p‑Akt/Akt between the API-2 group and the API-2+ drug + A/R group. This result indicates that API-2 can block the process when drug-containing serum inhibits autophagy. API-2 is an inhibitor of the Akt signaling pathway, indicating that drug-containing serum also inhibits autophagy through the Akt signaling pathway. The results are shown in Fig. 7.

## Discussion

Autophagy, an emerging research topic in recent years, has been found to play a key role in the occurrence and development of HF. Under normal conditions, autophagy occurs in the cardiac muscle of the human body. In the hypertrophic and ischemic state, autophagy protects against the damage of the body’s own cells by increasing their number. Studies have shown that, on one hand, autophagy is an adaptive response that protects the heart and prevents cell death caused by ischemia [8, 9]. On the other hand, when autophagy is excessive, it will accelerate the death of cardiomyocytes. Therefore, it is of great importance to study the mechanism of autophagy in cardiomyocytes and the role of ventricular remodeling after myocardial infarction. The proper regulation of autophagy can reduce heart remodeling and improve heart function. The occurrence of autophagy requires the intervention of autophagy transduction pathway. The PI3K/Akt/mTOR transduction pathway is used as the classic conduction pathway in autophagy. It was confirmed that by controlling the pathway, excessive autophagy could be controlled and myocardial remodeling would be improved and reversed [10–13].

In vivo experimental results in our study showed that NT-proBNP, ST2, IL-6, and TNF-α increased significantly in the model group compared with the sham group, suggesting that the HF model was successfully replicated. Typhaneoside treatment significantly reduced ST2 and IL-6. The expression of MMP-2 and MMP-9 increased significantly in the model group, and the HF model was successfully replicated. Compared with the model group, treatment reduced the levels of MMP-2 and MMP-9, suggesting that typhaneoside could improve myocardial remodeling by inhibiting the expression of MMP-2 and MMP-9.

In the present study, the autophagosomes of each group were observed by transmission electron microscopy. Compared with the control group, a large number of bilayer membrane autophagosomes were observed in the model group. Compared with the model group, the autophagosomes were significantly reduced in the drug treatment group. This indicates that after hypoxia/reoxygenation of primary cardiomyocytes, the autophagosomes increased and the modeling was successful, and thus pretreatment with the drug could inhibit autophagy during hypoxia/reoxygenation. To further validate the mechanism of the drug on autophagy in primary cardiomyocytes during hypoxia/reoxygenation, fluorescence imaging was performed and autophagosomes were examined by immunofluorescence. It was found that a large number of red spots appeared in the cytoplasm of the model group compared with the control group, indicating that after the hypoxia/reoxygenation of the primary cardiomyocytes, a large number of autophagosomes were formed, meaning the modeling was successful. In comparison, the reduction of red spots in the drug group was significant, suggesting that pretreatment with the drug intervened in the autophagy activity of the hypoxia/reoxygenation process and reduced the formation of autophagosomes.

Usually, LC3 II is considered a marker of autophagosomes in the early induction of autophagy. When it is upregulated, it means autophagy has been activated. In our immunoblotting experiment we found that, compared with the model group, the ratio of LC3-II/LC3-I in the RA + A/R group increased, and the expression of the p62 protein decreased significantly. Compared with the drug group, the ratio of LC3-II/LC3-I in the RA + drug + A/R group increased significantly, and the expression of the p62 protein decreased significantly. Compared with the RA + A/R group, the LC3-II/LC3-I ratio and the p62 protein expression in the RA + drug + A/R group did not change significantly, indicating that the inhibition of Mtorc1 by rapamycin affected the drug inhibition of autophagy. The inhibition of autophagy by drug serum (DS) in the cardiomyocyte hypoxia/reoxygenation process is associated with mTOR. Compared with the control group, the p‑mTOR/mTOR ratio in the model group increased significantly. Compared with the model group, the ratio of p‑mTOR/mTOR in the drug + A/R group was further improved. Compared with the model group, the p‑mTOR/mTOR ratio in the RA group decreased significantly. Compared with the RA group, the ratio of p‑mTOR/mTOR in the RA + drug + A/R group decreased significantly, indicating that DS could not reactivate mTOR proteins after RA inhibited mTOR. These results showed that RA can block the autophagy inhibition of DS during the cardiomyocyte hypoxia/reoxygenation /R process, which indicates that the drug-containing serum inhibits autophagy through the mTOR pathway.

In the API-2 groups, the level of autophagy in the API-2 + A/R group was further increased, the ratio of LC3-II/LC3-I was increased, and the expression of p62 protein was significantly decreased compared with the model group. In the API-2+drug + A/R group, the LC3-II/LC3-I ratio increased significantly, and the p62 protein expression decreased significantly. Compared with the control group, the ratio of p‑Akt/Akt in the model group increased significantly. Compared with the model group, the ratio of p‑Akt/Akt in the drug + A/R group was further improved. Compared with the A/R group, the ratio of p‑Akt/Akt the API-2 group was significantly decreased. Compared with the drug + A/R group, the ratio of p‑Akt/Akt in API-2 + drug + A/R group significantly decreased. In the API-2 and API-2+ drug + A/R groups, there was no obvious change in the p‑Akt/Akt ratio. These results indicate that API-2 can block the process when the drug-containing serum inhibits autophagy. API-2 is an inhibitor of the Akt signaling pathway, indicating that drug-containing serum also inhibits autophagy via Akt signaling pathway.

## Conclusion

In summary, typhaneoside can improve the morphological structure of the heart in a rat model of heart failure (HF) after myocardial infarction, can improve cardiac function indexes, and can improve myocardial remodeling, which may be related to the reduction of inflammatory factors and myocardial matrix metalloproteinase levels. Typhaneoside can reduce the level of autophagy in rats with HF after myocardial infarction. Furthermore, it can regulate the PI3K/Akt/mTOR transduction pathway by increasing the phosphorylation levels of Akt and mTOR, thereby inhibiting excessive autophagy and improving HF. Typhaneoside can inhibit the excessive autophagy of hypoxia/reoxygenation cells and regulate the phosphorylation of Akt and mTOR, suggesting that the inhibition of the PI3K/Akt/mTOR transduction pathway may be the mechanism by which typhaneoside inhibits autophagy.

## References

[1] Dhingra R, Margulets V, Chowdhury SR (2014). Bnip3 mediates doxorubicin induced cardiac myocyte necrosis and mortality through changes in mitochondrial signaling [J]. Proc Nat Acad Sci.

[2] Ling H, Chen H, Wei M (2015). The effect of autophagy on inflammation cytokines in renal ischemia/reperfusioninjury [J]. lnflammation.

[3] Zhu Y, Pires KM, Whitehead KJ (2013). Mechanistic target of rapamycin is essential formurineembryonic heart development and growth [J]. PLOS One.

[4] Orogo AM, Gustafsson AB (2015). Therapeutic targeting of autophagy:potential and concerns in treating cardiovascular disease [J]. Circ Res.

[5] Fullgrabe J, Klionsky DJJoseph B (2013). The return of the nucleus:transcriptional and epigenetic control of autophagy [J]. Nat Rev Mol Cell Bio.

[6] Nirmala H, Yoshiyuki I, Chull H, et (2013). Autophagy plays an essential role in mediating regression of hypertrophy during unloading of the heart [J]. PLOS One.

[7] Cao Y, Tao L, Shen S (2013). Cardiac ablation of Rhebl induces impaired heart growth,endoplasmic reticulum associated apoptosis and heart failure in infant mice [J]. Int J Mol Sci.

[8] Dhingra R, Margulets V, Chowdhury SR (2014). Bnip3 mediates doxorubicin-induced cardiac myocyte necrosis and mortality through changes in mitochondrial signaling [J]. Proc Nat Acad Sci.

[9] Zhu Y, Pires KM, Whitehead KJ (2013). Mechanistic target of rapamycin(Mtor)isessential for murineembryonic heart development and growth [J]. PLOS One.

[10] Orogo AM, Gustafsson AB (2015). Therapeutic targeting of autophagy:potenti-al and concerns in treating cardiovascular disease [J]. Circ Res.

[11] De Meyer GR, Grootaert Michiels MSCF (2015). Autophagy in vascu-lar disease [J]. Circ Res.

[12] Mei Y, Thompson MD, Cohen RA (2015). Autophagy and oxidative stress in cardiovascular diseases [J]. Biochim Biophys Acta.

[13] Sergin I, Bhattacharya S, Emanuel R (2016). Inclusion bodies enric-hed for p62 and polyubiquitin ated proteins in macrophages rotect against atherosclerosis [J]. Sci Signal.

